# An individually-tailored smoking cessation intervention for rural Veterans: a pilot randomized trial

**DOI:** 10.1186/s12889-016-3493-z

**Published:** 2016-08-17

**Authors:** Mark W. Vander Weg, Ashley J. Cozad, M. Bryant Howren, Margaret Cretzmeyer, Melody Scherubel, Carolyn Turvey, Kathleen M. Grant, Thad E. Abrams, David A. Katz

**Affiliations:** 1 Comprehensive Access & Delivery Research and Evaluation (CADRE) Center, Iowa City VA Health Care System, Mail Stop 152, 601 Highway 6 West, Iowa City, IA 52246 USA; 2 Department of Medicine, University of Iowa, Iowa City, USA; 3 Department of Psychological and Brain Sciences, University of Iowa, Iowa City, USA; 4 Veterans Rural Health Resource Center - Central Region, Iowa City, USA; 5 Department of Social Work, University of Iowa, Iowa City, USA; 6 Department of Psychiatry, University of Iowa, Iowa City, USA; 7 Mental Health and Behavioral Sciences Department, VA Nebraska-Western Iowa Health Care System, Omaha, USA; 8 University of Nebraska Medical Center Department of Medicine, Omaha, USA; 9 Department of Epidemiology, University of Iowa, Iowa City, USA

**Keywords:** Tobacco, Nicotine dependence, Smoking cessation, Veterans, Rural health

## Abstract

**Background:**

Tobacco use remains prevalent among Veterans of military service and those residing in rural areas. Smokers frequently experience tobacco-related issues including risky alcohol use, post-cessation weight gain, and depressive symptoms that may adversely impact their likelihood of quitting and maintaining abstinence. Telephone-based interventions that simultaneously address these issues may help to increase treatment access and improve outcomes.

**Methods:**

This study was a two-group randomized controlled pilot trial. Participants were randomly assigned to an individually-tailored telephone tobacco intervention combining counseling for tobacco use and related issues including depressive symptoms, risky alcohol use, and weight concerns or to treatment provided through their state tobacco quitline. Selection of pharmacotherapy was based on medical history and a shared decision interview in both groups. Participants included 63 rural Veteran smokers (mean age = 56.8 years; 87 % male; mean number of cigarettes/day = 24.7). The primary outcome was self-reported 7-day point prevalence abstinence at 12 weeks and 6 months.

**Results:**

Twelve-week quit rates based on an intention-to-treat analysis did not differ significantly by group (Tailored = 39 %; Quitline Referral = 25 %; odds ratio [*OR*]; 95 % confidence interval [*CI*] = 1.90; 0.56, 5.57). Six-month quit rates for the Tailored and Quitline Referral conditions were 29 and 28 %, respectively (*OR*; 95 % *CI* = 1.05; 0.35, 3.12). Satisfaction with the Tailored tobacco intervention was high.

**Conclusions:**

Telephone-based treatment that concomitantly addresses other health-related factors that may adversely affect quitting appears to be a promising strategy. Larger studies are needed to determine whether this approach improves cessation outcomes.

**Trial registration:**

ClinicalTrials.gov identifier number NCT01592695 registered 11 April 2012.

**Electronic supplementary material:**

The online version of this article (doi:10.1186/s12889-016-3493-z) contains supplementary material, which is available to authorized users.

## Background

Although the prevalence of cigarette smoking among adults in the United States (US) continues to decline, rates remain elevated in certain groups including military Veterans [1] and those living in rural areas [2]. The reasons for greater tobacco use in rural communities are complex and likely include a combination of personal characteristics (e.g., higher prevalence of low socioeconomic status) and social or community-related factors such as targeted marketing by the tobacco industry, economic dependence on tobacco growing in certain farming communities, greater social acceptance of smoking, and lax policies restricting smoking in private and public locations [2–4]. Health care-associated factors including limited access to treatment also appear to contribute [3, 4]. Being uninsured [4, 5], infrequent visits to and/or lack of a personal physician [4, 5], a lower likelihood of being counseled about tobacco use [6], and insufficient knowledge about available treatment resources also likely play a role [4, 7]. Considering that approximately 5.3 million Veterans, representing 24 % of all Veterans living in the US, reside in rural areas [8], this represents a substantial group of individuals who are at increased risk for smoking-related health problems.

Tobacco quitlines are often advocated as a strategy to improve treatment access. Proactive telephone counseling is efficacious [9] and cost-effective [10]. Unfortunately, these services are underutilized, with only 1–5 % of eligible smokers receiving this form of treatment [11]. Many referred patients cannot be reached or are reluctant to enroll in quitline counseling [12], suggesting that additional efforts are needed to connect smokers with treatment resources.

There are also several health-related factors that may adversely affect cessation outcomes once patients are enrolled in treatment including depressive symptoms [13], risky alcohol use [14], and concerns about weight gain [13–17]. However, standard cessation interventions are typically not designed to treat these concerns, leading for calls to develop strategies that more effectively address these issues [13–17].

Traditionally, depression, alcohol use, and weight management have been addressed independently of tobacco use, often using behavioral health and nutrition specialists. Unfortunately, requiring separate treatment by specialists for each of these conditions may exacerbate problems related to access for rural smokers. Further, such efforts tend to be poorly coordinated, resulting in fragmented care that does not take advantage of the synergies and increased efficiencies provided by addressing these issues in an integrated fashion [18]. Coordinated treatment models led by a care manager and in which mental health care experts serve as consultants to primary care have been shown to improve outcomes in patients with depression [19, 20]. However, such approaches have not been adequately evaluated in the context of treatment for tobacco use. Further, evidence suggests that this model may not be as effective in small rural primary care practice settings which do not have on-site mental health care providers [21].

Taken together, these findings suggest that comorbid issues related to depressive symptoms, risky alcohol use, and weight concerns are common among smokers and may interfere with quitting, but are not typically addressed in treatment. The goals of this pilot trial were to determine the feasibility and potential efficacy of an individually-tailored cessation intervention for rural Veteran smokers that simultaneously addressed these issues.

## Methods

The study was a randomized controlled pilot trial of two treatment approaches (tailored telephone counseling to address cigarette smoking and concomitant depressive symptoms, risky alcohol use, and/or weight concerns [Tailored] vs. referral to the participants’ state tobacco quitline [Quitline Referral]). The primary outcome was 7-day point prevalence abstinence at 12 weeks and 6 months after participants’ target quit dates.

### Participants

Participants included rural Veteran daily cigarette smokers aged ≥ 18 years who were interested in quitting and received care at a Midwestern VA Medical Center. Individuals were excluded if they did not have telephone access, were unable to provide informed consent, or did not have a stable residence. Those with a history of dementia or other significant cognitive impairment or a serious mental illness that would interfere with their ability to participate in phone counseling (e.g., catatonic or disorganized schizophrenia) were also excluded based on diagnoses contained in the electronic medical record (EMR). We also excluded patients with markedly elevated depressive symptoms and/or suicidal ideation (see below).

### Procedures

Potentially eligible participants were identified from the EMR based on (a) documented cigarette use or (b) recent (within 2 years) prescription for nicotine replacement therapy (NRT), bupropion, or varenicline and (c) an address indicating rural residence, determined using Rural–urban Commuting Area codes based on zip code [22]. Patients meeting basic eligibility criteria were sent a letter offering them participation in the trial, to which they could respond by returning a self-addressed postcard or contacting study staff by phone. Those expressing interest were mailed an informed consent document and baseline questionnaire, which included screening items to assess for eligibility for the supplemental behavioral counseling modules (described below). Upon their return, participants were randomly assigned to treatment conditions based on a computer-generated algorithm on a 1:1 allocation ratio using simple randomization without blocking. The computerized random allocation sequence was generated by the study data manager. Participants were enrolled and informed of their treatment assignment by the Project Coordinator (AJC). The study was approved by the University of Iowa Institutional Review Board. Reporting of the trial conforms to CONSORT 2010 Guidelines.

### Interventions

#### Quitline referral

Participants assigned to the comparison condition were referred via fax by the Project Coordinator (AJC) to the tobacco quitline for their state of residence. Quitlines subsequently contacted participants to initiate treatment. In order to best approximate standard care, no special arrangements were made with the quitlines to modify their treatment protocol.

#### Tailored tobacco intervention

The core component of the Tailored intervention, which was consistent across all participants in this condition, was based on standard cognitive behavioral approaches involving problem solving and coping skills training. The intervention was delivered by phone over six sessions lasting approximately 20–30 min each and consisted of four phases: 1) preparing to quit; 2) going through the quitting process; 3) maintaining short-term abstinence; and 4) relapse prevention [23]. Further details about the smoking cessation intervention are provided in an additional table [Additional file 1]. Calls were conducted approximately 1 week apart, although the timing could be adjusted to accommodate Veterans’ schedules.

As noted above, the baseline survey included items to screen for conditions that have been associated with a reduced likelihood of cessation. These included elevated depressive symptoms, risky alcohol use, and concerns about weight gain. Elevated depressive symptoms were determined using the Patient Health Questionnaire (PHQ-9) [24] based on scores of 5–19; those who scored > 20 and/or who endorsed suicidal ideation were referred to their primary care or mental health care provider, as appropriate, and were excluded from the trial. Risky alcohol use was identified based on a score of ≥ 4 on the Alcohol Use Disorders Identification Test (AUDIT-C) [25]. Concerns about post-cessation weight gain were determined based on a score of >5 on a 10-point scale ranging from 1 (not at all) to 10 (most ever) [26]. Participants in the Tailored condition could be eligible for anywhere from zero to three of the supplemental treatment modules, but participation in each was voluntary and left up to the individual. Counseling related to each of the supplemental modules was delivered concurrently with the smoking cessation intervention. Each module is briefly described below, with further details provided in Additional file 1.

#### Mood management

The intervention for elevated depressive symptoms was based on behavioral activation (BA) treatment for depression [27]. BA is a brief, structured approach that focuses on increasing exposure to rewarding activities that are consistent with one’s values and life goals. In a prior study, BA was associated with higher quit rates and greater reductions in depressive symptomatology relative to standard cessation treatment [28]. The BA approach used in the present study was adapted from the work of Lejuez and colleagues [27].

#### Alcohol risk reduction

The alcohol intervention followed VA/Department of Defense Clinical Practice Guidelines [29] for addressing risky alcohol use along with recommendations from the National Institute on Alcohol Abuse and Alcoholism. Participants were encouraged to adopt low risk drinking patterns (i.e., ≤ 2 drinks/day for men and ≤ 1 drink/day for women) but were allowed to set their own goals [30]. Participants who demonstrated evidence of alcohol abuse or dependence, based on a score of > 8 on the AUDIT-C, or who scored ≥ 4 (for men) or ≥ 3 (for women) on the AUDIT-C and reported a history of prior alcohol treatment, were offered referral to the hospital’s substance abuse treatment program but were allowed to continue with the study and intervention.

#### Weight management

The weight management intervention was based on a self-regulation approach [31] and focused on accepting and minimizing the weight gain that typically accompanies smoking cessation rather than targeting weight loss. Small, manageable changes in diet and physical activity were encouraged [32, 33]. Prior to initiating physical activity, participants were screened using the Physical Activity Readiness Questionnaire [34].

Interventions were delivered by a Ph.D. level social worker with expertise in substance abuse and a masters level counselor. Interventionists underwent training consisting of seven didactic sessions (lasting 1–1.5 h each) with the principal investigator (MWV). These sessions included a study overview, the smoking cessation counseling protocol, pharmacotherapy, medication selection, and each of the supplemental counseling modules. Prior to interacting with participants, interventionists practiced treatment with a standardized patient, who provided feedback on their delivery. In addition, the PI and interventionists met weekly to review participants and discuss treatment strategies using a case management approach. A small number of calls (~5 %) were randomly selected for review by the PI for purposes of assessing fidelity and providing feedback.

#### Pharmacotherapy

The approach to pharmacotherapy was the same for both groups. Medication options were based on the Clinical Practice Guideline [35] and the VA formulary and included several forms of NRT (patch, gum, lozenge), bupropion, and varenicline. Combination therapy was also available as appropriate. An initial review of participants’ EMRs was conducted by a study nurse to identify potential contraindications related to the medications as well as to review prior history of pharmacotherapy for smoking cessation. Results were summarized in an electronic grid which highlighted precautions and contraindications for each medication, along with other pertinent health information. The study physician (DAK) reviewed the grid and the participant’s EMR as needed and identified medications for which the participant was eligible.

Considering that limited information is available regarding which medication is likely to be most effective for a given patient, study staff then conducted a shared decision interview with participants to arrive at their medication preference. The interviewer engaged the participant in a discussion of prior experiences with smoking cessation medications and solicited ratings regarding the importance of eight separate medication attributes. This information was used to help the participant choose a medication. Those who selected varenicline or bupropion completed a brief screen for hopelessness and suicidal/homicidal ideation. For participants selecting bupropion, additional queries were made to assess for potential contraindications (e.g., history of seizures or head trauma) to supplement information obtained from the EMR. Participants’ medication choices were communicated electronically to the study physician and a standard 12-week supply of pharmacotherapy was mailed from the pharmacy. As required by VA policy, participants receiving varenicline were screened over the phone for neuropsychiatric side effects every 28 days while on the drug. Participants were allowed to request a change in their medication one time due to perceived lack of efficacy or side effects. Pharmacotherapy was provided free-of-charge.

### Measures

Measurements were obtained at baseline and at 12 weeks and 6 months after participants’ target quit dates. Follow-up data were collected through phone interview by study staff who were blinded to participants’ treatment conditions.

#### Primary outcome

Cessation was determined based on self-reported 7-day point prevalent abstinence (PPA). To meet criteria for abstinence, participants had to report no tobacco or e-cigarette use during the prior 7 days.

#### Exploratory outcomes

The frequency of depressive symptoms over the prior 2 weeks was measured using the PHQ-9 [24]. The PHQ-9 has good sensitivity and specificity with regard to identifying those with major depression, and is a reliable and valid measure of severity of depressive symptoms [24].

Alcohol use over the previous week was assessed using the 7-day Alcohol Timeline Follow-back Calendar (TFLC) [36]. The TFLC has been found to possess adequate test-retest reliability [37] and good validity when compared to collateral report [38].

Dietary intake was assessed using the Starting the Conversation (STC) questionnaire, an eight-item dietary assessment tool designed for clinical practice [39]. The STC assesses the frequency with which various foods were consumed over the past few months including fruit, vegetables, soda, fast food meals or snacks, and added fats. Despite its brevity, the STC has demonstrated temporal stability, adequate construct validity, and sensitivity to change following dietary intervention [39].

Physical activity level was measured using the Rapid Assessment of Physical Activity (RAPA) [40]. The RAPA assesses the level and intensity of physical activity as well as strength and flexibility training. The RAPA has demonstrated good criterion validity based on correlations with established activity measures, and discriminates well between those who do versus do not engage in regular activity [40].

#### Quitline counseling

For participants in the Quitline Referral condition, contact by their state quitline, enrollment in counseling, and the number of calls completed were assessed by self-report.

#### Descriptive variables

Additional measures were obtained to characterize the sample. Sociodemographic variables included age, sex, race and ethnicity, education, marital status, employment, and household income. Health-related items included self-reported height, weight, and self-rated health. Participants were also asked about their history of tobacco use, prior quit attempts lasting ≥ 24 h, and the presence of other smokers in their household. Motivation to quit smoking and to change drinking patterns was assessed using the Contemplation Ladder [41]. Nicotine dependence was measured using the Fagerström Test for Nicotine Dependence (FTND) [42].

### Sample size

An initial sample size of 50 participants (*n* = 25 per group) was determined to be adequate to evaluate the feasibility of the tailored tobacco intervention approach. Because enrollment accrued more quickly than anticipated, a total of 63 rural smokers were ultimately enrolled and randomized to treatment conditions.

### Data analysis

Baseline group comparisons were conducted using chi-square tests and independent samples t-tests. Twelve-week and six-month quit rates were compared using binary logistic regression. Because of the small sample size and missing data for some variables, multivariable regression analysis was not performed. The primary analysis was based on an intention-to-treat/penalized imputation approach in which participants who were lost to follow-up were assumed to be smoking. Results from complete case analyses are also presented.

Exploratory analyses examined the impact of the supplemental behavioral interventions on depressive symptoms, weight, alcohol use, and dietary intake using repeated measures analysis of variance (ANOVA). These analyses were restricted to participants in the Tailored condition who were eligible for and opted to receive the associated supplemental module and participants in the Quitline Referral condition who would have met eligibility criteria for the supplemental intervention if they had been randomly assigned to the Tailored condition. Given the small sample sizes, statistical power to detect group differences on these outcomes was limited; nevertheless, results are presented for descriptive purposes.

Due to insufficient variation in RAPA scores, the assumptions of repeated measures ANOVA were not met for physical activity outcomes. Therefore, these data were dichotomized for purposes of analysis according to whether or not participants met recommended levels of aerobic activity. Chi-square tests were used to compare the proportion of participants in each group who met activity recommendations at each time point.

## Results

**Fig. 1:**
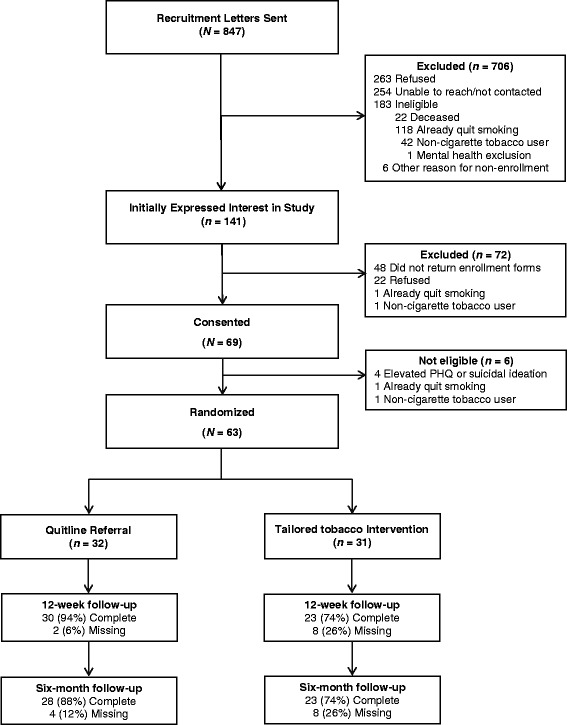
Enrollment and retention

Participants were enrolled in the study between June and November of 2012. The flow of participants through the study is presented in Fig. 1. Recruitment letters were mailed to 847 rural Veterans, of whom 706 were excluded. The main reasons for exclusion included refusal (*n* = 263), inability to contact (*n* = 60), not being reached prior to the end of the recruitment period (*n* = 194), and not meeting initial eligibility criteria (*n* = 183). Sixty three participants were randomized to treatment conditions (Tailored = 31; Quitline Referral = 32).

Baseline characteristics are presented in Table 1. Participants smoked an average of 24.7 (19.4) cigarettes/day and had a mean FTND score of 5.7 (2.2). Readiness to quit smoking averaged 6.6 (1.2) on a scale of 1 to 10. Approximately 30 % of participants at least partially attributed a prior relapse to depressed mood (data not shown). Alcohol and increased appetite/weight gain were identified as contributors to prior relapse by 21 and 10 %, respectively. No significant differences in baseline characteristics were observed between groups.

**Table 1:** Participant characteristics

Variable	Quitline Referral	Tailored intervention	Total
	(*n* = 32)	(*n* = 31)	(*N* = 63)
Sociodemographics			
Age	58.5 (8.8)	55.1 (11.5)	56.8 (10.3)
Range	31–73	32–73	31–73
Female, n (%)	5 (15.6)	3 (9.7)	8 (12.7)
Racial or ethnic minority, n (%)	2 (6.3)	1 (3.2)	3 (4.8)
High school or less education, n (%)	15 (46.9)	16 (51.6)	31 (49.2)
Fair or poor self-rated health, n (%)	15 (46.9)	13 (41.9)	28 (44.4)
Married, n (%)	15 (46.9)	15 (48.4)	30 (47.6)
Employed for wages outside home, n (%)	6 (19.4)	10 (35.7)	16 (27.1)
Annual household income < $25,000, n (%)	19 (59.4)	12 (42.9)	31 (51.7)
Smoking-related variables			
Cigarettes smoked per day	27.2 (25.7)	22.3 (10.3)	24.7 (19.4)
Age of smoking initiation	15.8 (2.8)	16.7 (3.4)	16.2 (3.1)
Years as regular smoker	40.9 (9.3)	36.5 (10.9)	38.6 (10.3)
Prior quit attempts lasting ≥ 24 h	9.4 (19.6)	6.6 (8.1)	8.0 (15.0)
Other smokers in household, n (%)	13 (40.6)	13 (41.9)	26 (41.3)
Nicotine dependence^a^	5.7 (2.1)	5.6 (2.3)	5.7 (2.2)
Readiness to quit smoking^b^	6.5 (1.3)	6.8 (1.1)	6.6 (1.2)

^a^Measured using the Fagerström Test for Nicotine Dependence [42]. Possible scores range from 0 to 10

^b^Measured on a scale of 1 (No interest in quitting) to 10 (Have quit and will never smoke again) using the Contemplation Ladder [41]

Data extracted from participants’ EMRs revealed a high frequency of chronic medical and mental health comorbidities; over 40 % rated their health as “fair” or “poor.” Thirty-eight percent had a documented history of cardiovascular disease, 22 % had chronic obstructive pulmonary disease, and 10 % had diabetes mellitus. With regard to mental health conditions, 22 % had a history of PTSD, 25 % had a history of an alcohol use disorder, and 32 % had a history of major depressive disorder (MDD). Marginally more participants in the Tailored condition had a history of MDD (42 % vs. 22 %), *X*^2^ (1) = 2.92, *p* = .087. None of the other comorbidities differed significantly by group.

### Screening results for supplemental behavioral interventions

Fifty-eight percent of participants assigned to the Tailored condition were eligible for the mood management intervention. The proportions meeting criteria for the alcohol risk reduction and weight management interventions were 40 and 23 %, respectively. Eighty-nine percent of eligible participants accepted the mood management intervention, while 33 and 100 % of those who were eligible accepted the alcohol and weight interventions, respectively. One additional participant who scored four on the weight concerns scale who was mistakenly offered the weight management module also accepted and received the intervention. No participants accepted a referral to the facility’s substance use treatment program. Overall, 16 participants (52 %) received one of the three supplemental treatment modules and six participants (19 %) received two. Nine participants (29 %) received only the smoking cessation intervention.

### Receipt of telephone counseling

Fifty-two percent of participants assigned to the Tailored intervention completed all six treatment calls. The overall attendance rate was 66 %, and the mean number of calls completed was 4.0. Five participants dropped out of the study prior to receiving any counseling. Sixty-seven percent of those in the Quitline Referral condition reported receiving a call from their state quitline. Of those who were contacted, 70 % enrolled in treatment, with a mean number of quitline counseling calls completed of 2.9 (*SD* = 1.4; range = 1 to 5).

### Choice of pharmacotherapy

Because the same procedure was followed for determining medications for both treatment conditions and considering that no group differences in medication selection were observed, results are summarized for the sample as a whole (Table 2). The most frequently chosen medication was the nicotine patch (48 %) followed by varenicline (37 %). Overall, 54 % selected some form of combination therapy. Eight participants (13 %) opted for monotherapy in the form of a single nicotine replacement product, while one participant (2 %) chose bupropion only. Varenicline was only available as monotherapy. Six participants switched medications during the course of treatment, including two in the Tailored condition (7 %) and four in the Quitline Referral condition (13 %). Two participants in the Tailored condition dropped out of treatment prior to receiving medication.

**Table 2:** Choice of pharmacotherapy by group

Medication^a^	Quitline Referral	Tailored intervention^b^	Total
	n (%)	n (%)	n (%)
Any use of product^c^			
Nicotine patch	18 (56.3)	12 (38.7)	30 (47.6)
Nicotine gum	10 (31.3)	7 (22.6)	17 (27.0)
Nicotine lozenge	9 (28.1)	9 (29.0)	18 (28.6)
Bupropion	8 (25.0)	5 (16.1)	13 (20.6)
Varenicline	10 (31.3)	13 (41.9)	23 (36.5)
Monotherapy			
Nicotine replacement therapy	6 (18.8)	2 (6.5)	8 (12.7)
Nicotine patch	2 (6.3)	0 (0.0)	2 (3.2)
Nicotine gum	3 (9.4)	0 (0.0)	3 (4.8)
Nicotine lozenge	1 (3.1)	2 (6.5)	3 (4.8)
Bupropion	1 (3.1)	1 (3.2)	2 (3.2)
Varenicline	10 (31.3)	13 (41.9)	23 (36.5)
Combination therapy			
Any combination NRT	12 (37.5)	11 (35.5)	23 (36.5)
Nicotine patch + nicotine gum	5 (15.6)	5 (16.1)	10 (15.9)
Nicotine patch + nicotine lozenge	7 (21.9)	6 (19.4)	13 (20.6)
Any bupropion + NRT combination	7 (21.9)	4 (12.9)	11 (17.5)
Nicotine patch + bupropion	4 (12.5)	1 (3.2)	5 (7.9)
Nicotine gum + bupropion	2 (6.3)	2 (6.5)	4 (6.3)
Nicotine lozenge + bupropion	1 (3.1)	1 (3.2)	2 (3.2)

^a^Participants were allowed to switch their medication choice one time due to perceived lack of efficacy or side effects. A total of six participants switched medications, including two participants in the Tailored intervention condition and four in the Quitline Referral condition. For those who switched medications, both choices are represented in the table

^b^Two participants in the tailored intervention condition dropped out of the intervention prior to choosing study medication

^c^As monotherapy or in combination with another medication

### Primary outcomes

Follow-up data collection occurred between August, 2012 and May, 2013. Self-reported 7-day PPA based on penalized imputation and complete case analysis are presented in Table 3. Twelve-week data were available for 74 % of those in the Tailored intervention group and 94 % of those assigned to Quitline Referral. Although quit rates tended to be higher in the Tailored group, no statistically significant differences in abstinence were observed based on either penalized imputation (*OR* = 1.90; *95 % CI*: 0.65, 5.57) or complete case analysis (*OR* = 3.00; *95 % CI*: 0.95, 9.49).

**Table 3:** 7-day Point prevalence abstinence outcomes by group

Outcome	Quitline Referral	Tailored intervention	*OR* (*95 % CI*)^a^
	(*n* = 32)	(*n* = 31)	
12-week follow-up			
Penalized imputation	25.0	38.7	1.90 (0.65, 5.57)
Complete case analysis^b^	26.7	52.2	3.00 (0.95, 9.48)
Six-month follow-up			
Penalized imputation	28.1	29.0	1.05 (0.35, 3.12)
Complete case analysis^c^	32.1	39.1	1.36 (0.43, 4.30)

^a^Unadjusted

^b^Based on 53 participants (*n* = 23 for tailored intervention condition and *n* = 30 for referral condition)

^c^Based on 51 participants (*n* = 23 for tailored intervention condition and *n* = 28 for referral condition)

Six month data were obtained for 74 and 88 % of those in the Tailored and Quitline Referral groups, respectively. As with the 12-week results, the odds of cessation based on either penalized imputation methods (*OR* = 1.05; *95 % CI*: 0.35, 3.12) or complete case analysis (*OR* = 1.36; *95 % CI*: 0.43, 4.30) did not differ significantly by group.

### Exploratory outcomes

Exploratory outcomes are presented in Additional file 2. Changes in depressive symptoms did not differ by group (*Group* x *Time* interaction, *F* [2, 26] = 1.38, *p* = 0.27). There was, however, a main effect for *Time*, *F* (2, 26) = 44.4, *p* < .001, with depressive symptoms decreasing from baseline in both groups. Dietary intake scores decreased (improved) from baseline for the sub-sample as a whole, *F* (2, 12) = 6.6, *p* = .012. Changes over time did not, however, differ by group, *F* (2, 12) = 1.0, *p* = 0.40. With regard to body weight, no significant main effects for *Group* or *Time* were noted, nor was the *Group x Time* interaction significant.

The proportion of participants in each group who met recommended levels of aerobic activity is also presented in Additional file 2. Although participants in both groups tended to be more active at 12 weeks and 6 months than at baseline, no significant differences between the Tailored and Quitline Referral conditions were noted at any time point.

### Treatment satisfaction

The majority of participants in both conditions “extremely/very much” liked that the intervention was delivered by phone (Tailored = 91 %; Quitline Referral = 71 %, *X*^2^ (1) = 2.53, *p* = .112). The proportion of participants who indicated that treatment was “extremely useful” or “very useful” was 74 % for the Tailored condition and 46 % for the Quitline Referral condition, *X*^2^ (1) = 2.74, *p* = .096. Forty-six percent of those in the Quitline Referral condition rated the intervention as “very/extremely” difficult, compared with 25 % of those in the Tailored condition, *X*^2^ (1) = 1.36, *p* = .244. Finally, 86 % of participants in the Tailored condition indicated that they liked that the treatment addressed multiple issues in the same intervention “extremely” or “very much.”

## Discussion

Results from this pilot trial support the feasibility of a tailored tobacco intervention approach delivered via telephone. The proactive recruitment strategy identified a sizeable number of smokers who were interested in receiving cessation treatment that simultaneously addressed health concerns previously associated with reduced quitting success/relapse, suggesting that smokers are generally receptive to receiving assistance with other health behaviors in the context of tobacco treatment. However, while 40 % of Veterans in the intervention group qualified for the alcohol risk reduction intervention, only 1/3 of these agreed to participate in the module. Rural Veteran smokers with risky alcohol use may be less amendable to alcohol reduction strategies than interventions that address mood or concerns about weight gain. Participants also reported a high level of satisfaction with the individually-tailored treatment approach.

Tailored tobacco counseling and referral to a state tobacco quitline were associated with comparable cessation outcomes at 12 weeks and 6 months. Although the odds of quitting were 90 % higher in the Tailored condition at 12-weeks, this difference was not statistically significant and was not maintained at 6 months. It may be, therefore, that standard tobacco quitline counseling is as effective as a tailored approach, although it should be noted that those assigned to the Quitline Referral condition received more intensive medication management tailored to their history and preferences that differs from what is typically provided through US tobacco quitlines. Self-reported 7-day PPA rates in both groups were encouraging overall and generally comparable to or slightly higher than reported in prior studies involving quitline counseling (e.g., [10, 43]). These quit rates are particularly promising given the sample’s high prevalence of medical and mental health comorbidities, heavy smoking, and socioeconomic disadvantage.

Exploratory analyses revealed no differences in depressive symptoms, weight, or dietary intake by treatment group. However, these results should be considered preliminary due to the small sample sizes. Conclusions regarding the effectiveness of either intervention approach at managing these issues and improving cessation outcomes will require further study.

Strengths of the study include the proactive recruitment strategy, focus on an underserved group of smokers (rural Veterans), patient-centered approach that included optional supplemental interventions to aid with smoking cessation and associated concerns, and the novel strategy for selecting pharmacotherapy using shared decision making. The broad eligibility criteria, which allowed the enrollment of patients with medical or mental health conditions who are typically excluded from smoking cessation studies, was another strength.

Limitations include the modest sample size, which precluded definitive comparisons of tobacco use and other outcomes by group. The reliance on self-reported measures of tobacco use, body weight, and alcohol intake is another limitation. Although self-reported smoking status is generally considered valid in the context of most observational and many types of intervention studies [44, 45] there is evidence that medical patients, particularly those with smoking-related illnesses, are more likely to misreport abstinence [44–46]. Considering that quitline users who are lost to follow-up are more likely to be smokers [47] and that a greater proportion of participants in the Tailored relative to Quitline Referral condition did not provide follow-up data, results of the complete-case analyses may have been biased in favor of the Tailored group. Data pertaining to treatment received from the tobacco quitline for those in the Referral condition was also based on self-report, and may therefore be subject to memory lapse or recall bias. It should also be noted that exploratory analyses were restricted to those who were eligible for and accepted the supplemental interventions in the Tailored condition (and who were therefore motivated to address these issues), but included all participants in the Quitline Referral group who met eligibility criteria. Consequently, these analyses may also have been biased in favor of the Tailored condition.

In addition, we did not have access to information regarding the receipt of counseling for chronic health conditions and mental health concerns among patients in the Quitline Referral condition. Considering that many tobacco quitlines attempt to at least partially tailor intervention content based on certain smoker characteristics or needs [48, 49], it is quite possible that some participants assigned to the Quitline Referral condition also received a form of tailored intervention. Unfortunately, we do not have data from the state tobacco quitlines about the intervention content provided to participants. Furthermore, the fact that participants in the Quitline Referral condition were also asked questions about mood, alcohol use, and weight concerns may also have prompted them to think about or otherwise address these issues even if they were not a formal part of their smoking cessation intervention. Measurement reactivity could also have prompted changes on these outcomes in the absence of additional intervention [50]. Consequently, differences in outcomes between groups that might otherwise have resulted from a tailored intervention approach may have been attenuated.

Additionally, although we obtained information regarding participants’ choice of pharmacotherapy and the prescriptions they were given, data pertaining to medication adherence was not collected. Although telephone is generally regarded as a valid and cost-effective mode of data collection [51–53], this approach may have adversely affected data quality, particularly among older and ill participants, due to increased response burden, diminished hearing, difficulty concentrating, and impaired recall [51, 53]. The fact that only 7 % of those sent recruitment letters were ultimately consented for the study also raises questions regarding the viability and potential reach of this approach. However, this enrollment rate compares quite favorably to community- and population-based smoking cessation studies, for which recruitment averages approximately 2 % [54]. Finally, as this was a study of rural Veterans who were primarily male and white, non-Hispanic, generalizability to other smokers is uncertain.

The lack of standardization regarding the receipt of the supplemental treatment modules and provision of pharmacotherapy may also be viewed as a limitation. Decisions regarding whether to receive treatment for depressive symptoms, alcohol use, and weight concerns were based on positive screens and self-selection. The choice of pharmacotherapy was also left up to the participant, although it was restricted to medications for which they were medically eligible as determined by the study physician. While this approach is admittedly inferior to more traditional clinical trials with regard to maximizing internal validity, it more accurately reflects the decisions facing patients and providers in routine practice, and better takes patients’ preferences into account.

## Conclusions

An individually-tailored tobacco cessation intervention which combined pharmacotherapy, behavioral counseling for smoking cessation, and supplemental treatments for related concerns was feasible and well-received by participants. Self-reported cessation rates were encouraging but did not differ significantly from referral to a tobacco quitline. Further work with larger samples is needed to determine whether shared decision making for medication selection and tailored counseling that addresses multiple risk factors for relapse is associated with better outcomes than standard smoking cessation treatment. Given the lack of differences in cessation outcome at 6 months, it may be that standard quitline counseling is equally effective and more easily disseminated on a large scale.

## Additional files

Additional file 1: Intervention components. Description of data: Table containing the intervention content for the smoking cessation counseling and supplemental behavioral modules. (DOCX 28 kb) Additional file 2: Exploratory outcomes by group. (DOCX 30 kb)

## Abbreviations

AUDIT-C: Alcohol Use Disorders Identification Test
BA: Behavioral activation
EMR: Electronic medical record
FTND: Fagerström Test for Nicotine Dependence
MDD: Major depressive disorder
NRT: Nicotine replacement therapy
PHQ-9: Nine-item Patient Health Questionnaire
PPA: Point prevalence abstinence
Quitline referral: State tobacco quitline referral condition
RAPA: Rapid Assessment of Physical Activity
STC: Starting the Conversation dietary screen
Tailored: Tailored tobacco counseling condition
TLFC: 7-day Alcohol Timeline Follow-back Calendar
