# Supplementary material for: An individually-tailored smoking cessation intervention for rural Veterans: a pilot randomized trial
Source: BMC Public Health. 2016 Aug 17;16:811. doi: 10.1186/s12889-016-3493-z (PMC4989380; doi:10.1186/s12889-016-3493-z)
Supplement: Additional file 1: — Intervention components. Description of data: Table containing the intervention content for the smoking cessation counseling and supplemental behavioral modules. (DOCX 28 kb) [file 12889_2016_3493_MOESM1_ESM.docx]

**Additional File 1. Intervention Components**

| **Smoking Cessation** |
| --- |
| Setting a quit date |
| Stimulus control |
| Eliciting social support |
| Managing nicotine withdrawal |
| Using self-rewards to reinforce quitting |
| Motivational enhancement |
| Associations between smoking and caffeine |
| Problem solving |
| Coping skills training |
| Surfing the urge |
| Cognitive restructuring |
| Medication adherence |
| Relapse prevention |
| **Mood Management** |
| Self-monitoring |
| Associations between smoking and negative affect |
| Values identification |
| Goal setting |
| Increasing engagement in rewarding and valued activities |
| Reducing behavioral/experiential avoidance |
| Associations between avoidance and smoking |
| Eliciting social support |
| **Alcohol Risk Reduction** |
| Education regarding standard drink sizes |
| Normative drinking patterns |
| Health risks associated with heavy alcohol use |
| Associations between smoking and alcohol consumption |
| Motivational enhancement |
| Goal setting |
| Tips for reducing intake |
| Stimulus control |
| Engaging in activities not association with alcohol consumption |
| Problem solving |
| Coping skills training |

**Additional File 1. Intervention Components, Continued.**

| **Weight Management** |
| --- |
| Education about the nature of post-cessation weight gain |
| Prior use of smoking as a weight control strategy |
| Adopting realistic expectations of weight change after quitting smoking |
| Acceptance of modest weight gain that typically accompanies cessation |
| Limiting postcessation increases in energy intake |
| Adopting small but meaningful changes in diet and activity |
| Promoting lifestyle activity |
| Benefits of regular activity for quitting smoking |
| Colorful eating as a strategy to improve nutritional quality of diet |
| Self-monitoring diet and activity |
| Stimulus control |
| Problem solving |
