# Supplementary material for: An individually-tailored smoking cessation intervention for rural Veterans: a pilot randomized trial
Source: BMC Public Health. 2016 Aug 17;16:811. doi: 10.1186/s12889-016-3493-z (PMC4989380; doi:10.1186/s12889-016-3493-z)
Supplement: Additional file 2: — Exploratory outcomes by group. (DOCX 30 kb) [file 12889_2016_3493_MOESM2_ESM.docx]

Additional File 2. Exploratory Outcomes by Group.

Table 4. Exploratory Outcomes by Group.

| **Tailored Tobacco Intervention** | | | | | | **Quitline Referral** | |  |
| --- | --- | --- | --- | --- | --- | --- | --- | --- |
|  |  | ***Baseline*** | ***12 weeks*** | ***6 months*** |  | ***Baseline*** | ***12 weeks*** | ***6 months*** |
| **Outcome** | n | ***Median (q^1^-q^3^)^a^*** | ***Median (q^1^-q^3^)^a^*** | ***Median (q^1^-q^3^)^a^*** | n | ***Median (q^1^-q^3^)*^a^** | ***Median (q^1^-q^3^)*^a^** | ***Median (q^1^-q^3^)*^a^** |
| Depressive symptoms^b^ | 13 | 12.0 (6.5-15.5) | 7.0 (2.0-10.0) | 6.0 (2.0-11.0) | 16 | 9.5 (6.0-14.8) | 6.0 (2.3-8.8) | 4.0 (2.3-5.8) |
| Self-reported weight (lbs) | 6 | 215.0 (163.8-244.3) | 221.5 (171.3-245.8) | 214.0 (171.5-253.0) | 9 | 228.0 (190.0-226.0) | 234.0 (194.0-261.3) | 235.0 (185.0-269.0) |
| Dietary intake^c^ | 6 | 7.5 (5.7-8.3) | 6.0 (5.0-7.0) | 6.5 (3.75-7.8) | 9 | 6.5 (2.5) | 5.6 (2.6) | 4.3 (2.3) |
| Physical activity^d^ | 6 | 3.5 (3.0-4.0) | 4.0 (3.3-4.0) | 4.0 (2.5-4.0) | 9 | 4.0 (3.5-4.0) | 4.0 (3.5-4.0) | 4.0 (4.0-4.0) |
| Regularly active, *n* (%)^e^ |  | 4 (50) | 5 (83) | 4 (67) |  | 7 (58) | 8 (73) | 8 (89) |
| Alcohol intake^f,g^ | 2 | 16.0 (--)^i^ | 7.0 (--)^i^ | 9.5 (--)^i^ | 4 | 7.5 (1.0-23.0) | 1.5 (0.0-6.8) | 2.5 (0.0-5.8) |
| Alcohol ladder^f,h^ | 2 | 4.0 (--)^i^ | 7.5 (--)^i^ | 2.5 (--)^i^ | 4 | 9.5 (1.0) | 6.3 (4.8) | 6.0 (4.2) |

^a^Interquartile range (IQR).

^b^Measured with the Patient Health Questionnaire – 9 (PHQ-9). Possible scores range from 0 to 27. Scores of 0-4 are considered indicative of minimal depressive symptoms, while scores of 5-9, 10-14, 15-19, and 20-27 indicate mild, moderate, moderately severe, and severe depressive symptoms, respectively.

^c^Measured with the Wisewoman Starting the Conversation dietary instrument. Possible scores range from 0 to 14, with lower scores reflecting healthier dietary patterns. Mean substitution was used to impute missing values for four participants (two in each condition) who had missing responses for one item.

^d^Measured with the Rapid Assessment of Physical Activity (RAPA) measure. Possible scores range from 0 (sedentary) to 4 (regularly active).

^e^Coded as a dichotomous variable contrasting those who were regularly active (defined as 30+ minutes/day of moderate physical activity 5+ days/week or 20+ minutes/day of vigorous physical activity 3+ days/week) and those who were sedentary or active at levels below physical activity guidelines. Because separate analyses were conducted for each time point, the number of participants per group varies across time.

^f^Because there were only two participants in the tailored intervention condition who completed follow-up and met criteria for inclusion in the alcohol analyses, data related to weekly alcohol consumption and readiness to change drinking patterns are presented descriptively without a formal test of group differences.

^g^Total alcohol consumption (in drinks) over the past seven days. Measured using the 7-day Alcohol Timeline Follow-back Calendar.

^h^Measured using a Readiness to Change Drinking ladder. Possible scores range from 0 (No thoughts of changing drinking) to 10 (Taking action to change drinking.

^i^IQR could not be calculated due to the small number of cases.
